# Apolipoprotein D in Lipid Metabolism and Its Functional Implication in Atherosclerosis and Aging

**DOI:** 10.18632/aging.100004

**Published:** 2008-12-12

**Authors:** German Perdomo, H. Henry Dong

**Affiliations:** Rangos Research Center, Division of Immunogenetics, Department of Pediatrics, Children's Hospital of Pittsburgh, University of Pittsburgh School of Medicine, Pittsburgh, PA 15213, USA

**Keywords:** ApoD, HDL, LDL, VLDL, Dyslipidemia, Obesity, Diabetes, Atherosclerosis, Aging

## Abstract

Dyslipidemia is characterized by increased triglyceride and low-density lipoprotein (LDL) levels, and decreased high-density lipoprotein (HDL) levels. Such an atherogenic lipid profile often predisposes an at risk individual to coronary artery disease with incompletely understood mechanisms. Apolipoprotein D (apoD) is an atypical apolipoprotein. Unlike canonical apolipoproteins that are produced mainly in liver and intestine, apoD is expressed widely in mammalian tissues. ApoD does not share significant degrees of homology in amino acid sequence with other apolipoproteins. Instead, apoD is structurally similar to lipocalins, a diverse family of lipid-binding proteins that are responsible for transporting lipids and other small hydrophobic molecules for metabolism. Plasma ApoD is present mainly in HDL and to a lesser extent in low density lipoproteins (LDL) and very low-density lipoproteins (VLDL). Genetic variants of apoD are associated with abnormal lipid metabolism and increased risk of developing metabolic syndrome. Increased apoD deposition is detectable in atherosclerotic lesions of humans with established cardiovascular disease as well as mice with premature atherosclerosis. Moreover, apoD is associated with anti-oxidation and anti-stress activities, contributing to lifespan expansion in fruit flies. Elderly subjects and patients with Alzheimer exhibit markedly elevated apoD production in the brain. Thus, apoD is emerged as a significant player in lipid metabolism and aging. Here we focus our review on recent advances toward our understanding of apoD in lipid metabolism and address whether apoD dysregulation contributes to the pathogenesis of dyslipidemia and atherosclerosis. We will also discuss the functional implication of apoD in aging.

## Atherosclerosis

Coronary artery disease (CAD) is the leading cause of death in America. It happens when the arteries that supply blood to cardiac muscle become hardened and narrowed. This is due to excessive deposition of cholesterol, fatty substances and cellular waste products in the inner lining of coronary artery. Such a pathological condition, termed atherosclerosis, can happen in men and women, particularly at a later age. Aside from genetic predisposition, factors that account for the risk of atherosclerosis include dyslipidemia, hypertension, obesity, diabetes and smoking. These factors alone or in combination can hasten the progression of atherosclerosis and development of CAD. Although progress has been made in elucidating the pathophysiology of atherosclerosis, the exact cause and mechanism underlying the development of atherosclerosis still remain obscure [1-3].

Cholesterol homeostasis plays an important role in atherosclerosis. Cholesterol is an essential component of cellular membrane and also a precursor for the synthesis of steroid hormones and bile acids. Cholesterol in the body derives from two different sources, dietary intake and *de novo* synthesis in tissue such as liver - the major organ for endogenous cholesterol supply. Unlike fatty acids and triglyceride, cholesterol cannot be catabolized. Excessive cholesterol must be rid itself of the body via its transportation to liver for biliary excretion. This pathway, termed "reverse cholesterol transport", is facilitated by high-density lipoprotein (HDL) and is viewed as the primary mechanism by which HDL protects against the development of atherosclerosis [4-7]. Clinical data and preclinical studies have conclusively demonstrated that lower HDL levels constitute an independent risk factor for coronary artery disease. However, the molecular basis underlying the cardioprotective action of HDL remains incompletely understood. For better clinical management of CAD, further studies are warranted to better understand cholesterol metabolism and pathogenesis of atherosclerosis.

Apolipoprotein D (apoD) is a component of HDL. Due to its relative low abundance in HDL particles, apoD has received considerably less attention in the research area of HDL-cholesterol metabolism and atherosclerosis. Recent data indicate that aberrant apoD expression is associated with altered lipid metabolism and risk of coronary artery disease. This has spurred us to conduct a comprehensive review of apoD function in triglyceride and cholesterol metabolism to address the question of whether apoD is another significant player in the pathogenesis of atherosclerosis.

## ApoD production in health and disease

In humans, plasma apoD levels range from 3 to 11 μmol/L. This level is equivalent to plasma levels (4.9±0.5 μmol/L) of apolipoprotein C-III (apoC-III), an important player in plasma triglyceride metabolism [8-11]. However, plasma apoD levels are upregulated under certain pathophysiological conditions, such as in women with gross cystic disease [12]. ApoD levels are also elevated in the brain of subjects with chronic schizophrenia and in the prefrontal cortex of patients with Alzheimer disease [13-15]. Furthermore, treatment with antipsychotic drugs, expecially clozapine, also results in elevated apoD expression in rodent brains as well as in human plasma [13,16,17]. Increased apoD production is seen in the rat brain following traumatic brain injury [18].

In addition, elevated apoD production is detected in liver tumors resected from hepatocellular carcinoma [19], as well as in invasive carcinoma of the breast [20,21]. Elevated apoD levels are present in cyst fluids of women with gross cystic disease of the breast [12]. Furthermore, increased apoD levels are also detected in the breast nipple aspirate fluid in women with breast cancer, but nipple fluid apoD levels do not seem to correlate with the stage of the breast cancer disease [22].

Recently, Rickhag et al. [23]  demonstrate in a rat model of stroke that apoD is significantly elevated in the peri-infarct area during the recovery period. It is suggested that upregulated apoD production serves the function of transporting cholesterol and phospholipids, a remodeling process that is required for the recovery of brain injury. Likewise, high apoD protein levels are found in patients with failing hearts, compared with non-failing control subjects, raising the possibility that apoD is potential biomarker in human end-stage heart failure [24].

Niemann-Pick Type C (NPC) disease is a human neurodegenerative disorder characterized by impaired intracellular cholesterol transport [25]. Interestingly, in rodent models of the human NPC disease, apoD protein levels are markedly elevated by 30-fold in the brain and 6-fold in plasma, correlating with increased intracellular cholesterol storage [26,27]. These findings implicate apoD in the pathogenesis of NPC disease.

In addition to its altered expression in the brain, apoD is upregulated in cultured myotubes from patients with type 2 diabetes [28]. Likewise, enhanced apoD expression is detected in muscle biopsies from patients with disuse atrophy, a pathological condition that impacts muscle function and activity of daily living [29]. Interestingly, the induction of apoD mRNA expression is accompanied by a corresponding increase in leptin receptor mRNA in the immobilized muscle of patients with disuse atrophy. Immunohistochemistry colocalizes apoD and leptin receptor in the perinuclear area in the immobilized muscle fibers [29]. These data are consistent with the observation of Liu et al. [30], who show that apoD and leptin receptor physically interact with each other in the brain. ApoD and leptin receptor expression are coordinately regulated in the hypothalamus in modulating food intake and energy homeostasis in mice. Dissociation of apoD with the leptin receptor is linked to the development of obesity in leptin receptor-deficient db/db mice [30].

## Posttranslational modification of apoD

**Figure 1.**
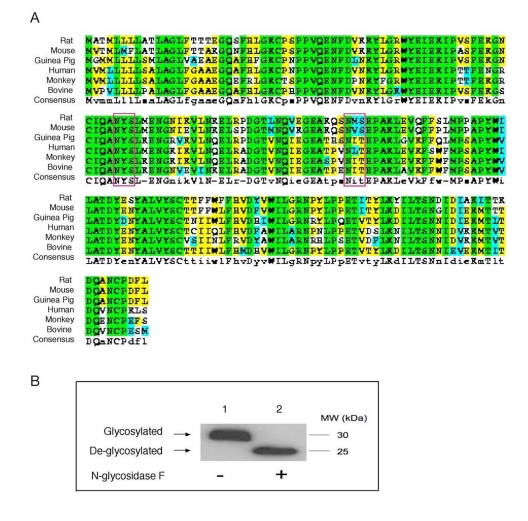
Conservation of N-glycosylation sites in apoD among species. (A). ApoD protein sequences of different species were aligned using the ClustalW program. Amino acid residues in box denote two highly conserved N-glycosylation sites in apoD. The consensus N-glycosylation site is Asn-X-Ser/Thr. **(B)** Plasma apoD is N-glycosylated. Aliquots of plasma (20 μg protein) from C57BL/6J mice were incubated without (-) and with (+) 1,000 U of N-glycosidase F (New England Biolabs) in a total volume of 30 μl at 37°C for 1 hour to remove N-glycan chains from glycopeptides. The reaction mixture was resolved on 4-20% SDS-polyacrylamide gels, followed by immunoblot analysis using anti-apoD. Glycosylated and de-glycosylated forms of apoD are indicated.

ApoD possesses two N-glycosylation sites (Asn45 and Asn78) [31], both of which are evolutionally conserved among species (Figure 1A). This raises the hypothesis that apoD is regulated at the post-translational level. In support of this hypothesis, we incubated aliquots of sera from normal C57BL/6J mice in the absence and presence of peptide:N-glycosidase F (PNGase F), an amidase that catalyzes the removal of carbohydrate moieties from N-linked glycoproteins. As shown in Figure 1B, pre-incubation of plasma apoD with PNGase F resulted in apoD de-glycosylation, as evidenced by the production of de-glycosylated apoD with reduced molecular masses. Likewise, serum apoD as well as apoD secreted from axillary glands in humans are also glycosylated [32,33]. While  the physiological  significance of this  post-translational modification remained incompletely understood, it is suggested that N-glycosylation modulates apoD protein folding, resulting in conformational changes favorable for binding to its physiological ligands or association with HDL. In this context, it would be of significance to convert the two N-glycosylation sites Asn45 and Asn78 to alanine residues in apoD by site-directed mutagenesis. The resulting apoD mutants will be ideal molecules for determining the physiological impact of N-glycosylation on the ability of apoD to associate with ligands and/or with HDL in metabolism.

## Effect of apoD on HDL-cholesterol metabolism

**Figure 2.**
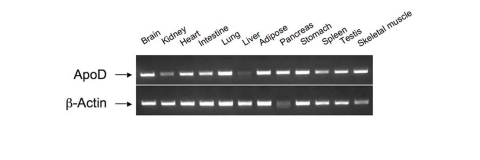
Tissue distribution of apoD mRNA expression. Total RNA was prepared from different tissues of C57BL/6J mice. Aliquots of purified RNA (100 ng) were subjected to RT-PCR analysis using apoD sequence-specific primers. The resulting PCR products were resolved on 1% agarose gel containing ethidiumbromide and visualized by UV light.

ApoD is an atypical apolipoprotein of 169 amino acids. Unlike canonical apolipoproteins that are produced mainly in liver and intestine, apoD is expressed widely in mammalian tissues including brain, liver, intestine, cardiac and skeletal muscle, adipose tissue, and pancreas [34-36] (Figure 2). ApoD does not share significant degrees of homology in the amino acid sequence with other apolipoproteins. Instead, apoD  is structurally similar to the lipocalin family of proteins. This superfamily comprises a diverse class of lipid-binding proteins including fatty acid binding proteins (FABPs), plasma retinol-binding proteins (RBP) and apolipoprotein M (apoM) [34,37-39]. Despite their dissimilarities in amino acid sequences, the lipocalin superfamily of proteins share a highly conserved β-barrel structure that is comprised of an eight-stranded anti-parallel β-sheet [40]. Such a tertiary architecture is predicted to form a ligand-binding pocket that is thought for binding and transporting lipids and other small hydrophobic molecules [34,37]. This characteristic lipocalin fold is validated for apoD by Eichinger et al. [41], who recently crystalized the human apoD protein in its free form and in complex with progesterone. Cystallographic studies reveal that the eight-stranded anti-parallel β-sheets of apoD are connected by four loops in a pair-wise manner, forming a conically shaped cavity that is capable of binding hydrophobic ligands [40,41]. Consistent with its structural organization, apoD is shown to associate with a number of ligands including cholesterol, progesterone, pregnenolone, bilirubin and arachidonic acid [13,34].

**Figure 3.**
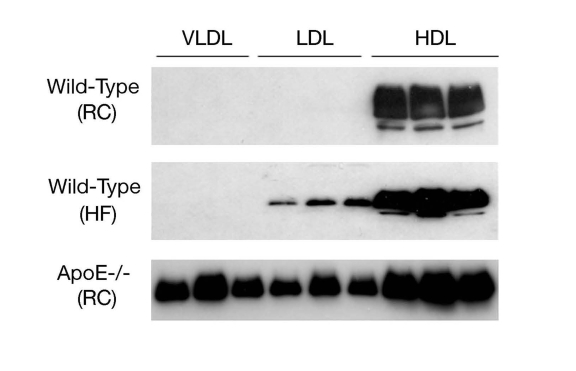
ApoD distribution in lipoproteins. Male C57BL/6J mice (3-5 weeks old) were fed regular chow (RC) or high fat diet (HF) for 8 weeks. Aliquots of 250-μl sera of mice (n=5) were fractionated by gel filtration column chromatography. Fractions (500 μl) were collected for the determination of cholesterol concentrations. Likewise, aliquots of sera (250 μl) of male apoE knockout mice (ApoE-/-, 12 weeks old on regular chow) were fractionated to VLDL, LDL and HDL fractions. Peak fractions of VLDL, LDL and HDL were subjected to immunoblot assay using anti-ApoD antibody.

Circulating ApoD is present mainly in HDL and to a lesser extent in LDL and VLDL [42,43](Figure 3). Nonetheless, little is known about the role of apoD in lipoprotein metabolism and its impact on atherosclerosis. Plasma apoD levels are significantly reduced in patients with Tangier disease, a rare autosomal recessive disorder that is caused by mutations in the ATP-binding cassette A1 (ABCA1) gene [34,44]. As ABCA1 plays a key role in effluxing cholesterol from cells, ABCA1 loss-of-function results in diminished cholesterol removal from peripheral tissues, contributing to excessive accumulation of cholesterol in the body and increased risk of developing atherosclerosis in patients with Tangier disease [45-50].

Recently, Vaisar et al. [51] took a proteomics approach to profile protein composition of HDL particles isolated from human subjects. Their studies reveal that HDL isolated from healthy individuals versus subjects with established CAD carry different protein cargos. Interestingly, apoD is highly enriched in HDL isolated from seven subjects with CAD, in comparison to six healthy controls. This observation seems paradoxical, as CAD patients are associated with lower HDL levels and apoD is mainly bound to HDL in the circulation. An increased apoD content in HDL may present a pathological marker or constitute a compensatory response to impaired cholesterol metabolism in subjects with established CAD.

**Figure 4.**
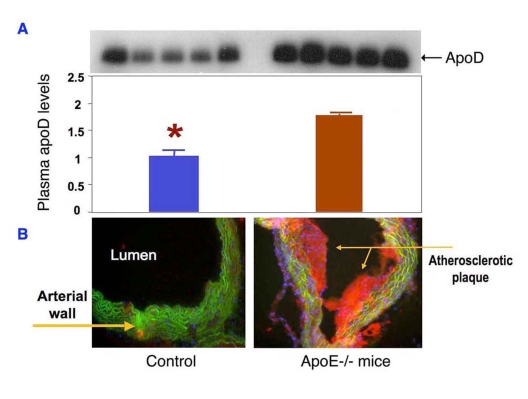
ApoD production is upregulated in atherogenic mice. **(A)** Sera of ApoE knockout (n=5) and control mice (n=5) at 12 weeks of age were subjected to immunoblot analysis using anti-apoD antibody. **(B)** Sections of aorta were stained by oil red O to visualize the atherosclerotic lesions in the aorta of apoE knockout mice. Data were obtained from 16-wk old mice. *P <0.05 vs. ApoE-/- mice by ANOVA.

To recapitulate this clinical observation, we determined plasma apoD expression in normal and atherogenic mice with genetic depletion of apolipoprotein E (apoE), a commonly used rodent model of atherosclerosis. ApoE knockout mice display a marked increase in total plasma cholesterol levels and develop atherosclerosis with the deposition of fatty streaks in the proximal aorta at 3 months of age. We show that plasma apoD levels are markedly increased in apoE knockout mice (Figure 4). These results together with clinical data presage a potential role of apoD in the pathogenesis of atherosclerosis.

## Abnormal apoD production in metabolic syndrome

In addition to its role in cholesterol homeostasis, apoD is involved in triglyceride metabolism. Epidemiological studies identified three distinct missense mutations, namely Phe36Val, Tyr108Cys and Thr158Lys in the apoD gene in African populations. Each of these three mutations is associated with significantly elevated plasma triglyceride levels and reduced HDL-cholesterol levels, a plasma lipid profile that is characteristic of metabolic syndrome [52,53]. Although the underlying molecular basis remains to be defined, these clinical data implicate abnormal apoD function in the pathogenesis of metabolic syndrome.

Consistent with this idea, two studies demonstrate a linkage between the *Taq*I polymorphism of the apoD gene and type 2 diabetes in South Indians and Nauruans [54,55]. Subsequently, Vijayaraghavan et al. [56] report that the *Taq*I polymorphism of apoD is associated with the development of obesity, insulin resistance and hyperinsulinemia in the British Caucasoid population. This effect seems to be independent of body weight, as no significant association is detected between the apoD polymorphism and body mass index (BMI) or waist to hip ratio in the same cohort of subjects [56].

Curry et al. [57] report that plasma apoD levels are significantly lower in patients with hyper-chylomicronemia. In a separate study to identify the factors that affect lipids and apolipoproteins at birth, Lane et al. [58] show that significant reductions in triglyceride and ApoD levels are detected in infants who subsequently became ill in the postnatal period with problems relating to carbohydrate metabolism (e.g., infants of diabetic mothers). Together these clinical data suggest that apoD is another significant player in lipid metabolism. ApoD dysregulation may contribute to metabolic abnormalities in insulin resistant subjects with obesity and/or type 2 diabetes.

Further evidence of apoD as a significant player in lipid metabolism derives from the studies in obese *db/db* mice. Due to leptin receptor deficiency, *db/db* mice are hyperphagic, developing morbid obesity and type 2 diabetes at about 12 weeks of age. Interestingly, Liu et al. [30] show that apoD and leptin receptor, which are co-expressed in the hypothalamus, interact with each other in regulating food uptake and body weight gain. Hypothalamic apoD mRNA is markedly induced in response to high fat feeding. However, this effect is abolished with a concomitant reduction of apoD mRNA levels in the hypothalamus of obese *db/db* mice. These data suggest that apoD may participate in the regulation of food intake and body fat accumulation via crosstalking with the leptin receptor.

## ApoD in HDL remodeling

There are two lines of evidences suggesting that apoD contributes to HDL remodeling. First, apoD is shown to modulate the activity of lecithin:cholesterol acyltransferase (LCAT), an HDL-bound enzyme that catalyzes the conversion of free cholesterol to cholesterol ester that is sequently recruited into the core of HDL. This effect along with apolipoprotein E (apoE) contributes to HDL core expansion and promotes HDL maturation [59]. Albers et al. report that apoD is a carrier of lysolecithin, a product of the LCAT reaction [60]. This finding is accordance with the observation that apoD interacts with LCAT [61]. However, whether apoD acts as an activator or inhibitor of LCAT activity still remains controversial. Studies by Kostner et al.[62] suggest that apoD is an activator of LCAT, which is at variance with the data of Albers et al. [63], who show that apoD is an inhibitor of LCAT. Steyrer et al. [64] studied the activation of LCAT activity by apoD in comparison to apoA-I and apoC-I in reconstituted proteoliposomes. ApoA-I is the most potent activator of LCAT, followed by apoC-I and apoD. Their studies suggest that apoD modulates LCAT activity presumbly by stabilizing the enzyme on HDL [64].

Second, apoD contributes to HDL remodeling via its covalent cross-link with apolipoprotein A-II (apoA-II), a structural component of HDL. Blanco-Vaca et al. [65] detect the presence of disulfide-linked heterodimers of apoD and apoA-II in human plasma. Non-reducing polyacrylamide gel electrophoresis demonstrates that the apoD-apoA-II heterodimer has an apparent molecular mass of 38 kDa, which is significantly larger than monomeric apoD (MW, 29 kDa). Sequence analysis reveals the presence of five cysteine residues in the human apoD protein. Mass Spectrometric analysis in combination with crystallographic studies of human apoD protein illustrates that four cysteines (Cys16-Cys41 and Cys8-Cys114) are primed for forming two intra-molecular disulfide bonds and the remaining unpaired cysteine (Cys-116) is responsible for inter-molecular covalent cross-link with Cys-6 of apoA-II within HDL [31,41]. Interestingly, the rodent apoD lacks the unpaired Cys-116, as it is replaced by threonine at the corresponding amino acid residue. Thus, the physiological significance of this covalent cross-link between apoD and apoA-II in HDL remodeling and cholesterol metabolism remains elusive [41].

## ApoD in oxidative stress and aging

Increased oxidative stress is closely associated with inflammation, insulin resistance, diabetes and atherosclerosis. There is accumulating evidence that apoD plays an important role in oxidative stress. Do Carmo et al. [66] show in cultured NIH/3T3 fibroblasts that apoD expression is significantly induced in response to cellular stress, regardless of whether the stress condition is caused by lipopolysaccharide (LPS) stimulation, H_2_O_2_ treatment or UV-light irradiation. This effect seems to be mediated by the NF-kB pathway, as there are several conserved NF-kB binding sites in the apoD promoter [66]. Furthermore, Do Carmo et al. [67]show that apoD confers a neuroprotective effect in the brain of mice. Their studies demonstrate that mice over-expressing human apoD inneurons, as opposed to normal controls, are more resistant with a 3-fold higher survival rate in response to human coronavirus-induced acute encephalitis. Likewise, Ganfornina et al. [68] show that apoD overexpression in the brain protects mice from oxidative stress. This effect correlates with the ability of apoD to prevent lipid peroxidation in cells [68].

Additional evidence of apoD function against oxidative stress stems from studies in fruit flies. Sanchez et al. [69] show that genetically modified *Drosophila* mutants with loss-of-function of the human apoD homolog gene (*GLaz*) exhibit high sensitivity to oxidative stress and nutrient deprivation. The *GLaz* mutant flies also have an increased accumulation of lipid peroxidation products in the body, accompanied by 10-15% reduction in lifespan. Conversely, Walker et al. [70]show that Drosophila with overexpression of the apoD homolog *GLaz *displays enhanced resistance to starvation and oxidative stress. ApoD overexpression also ameliorates lipid peroxidation with a 30% extension of lifespan in flies [70]. Similar observations are made by Muffat et al. [71], who demonstrate that overproduction of the human apoD are also associated with significantly reduced lipid peroxidation products, protecting against oxidative stress and extending lifespan by about 40% in fruit flies. Together these data demonstrate an evolutionally conserved safeguarding mechanism by which apoD acts to protect against lipid peroxidation and oxidative stress.

Although apoD is shown to confer a significant beneficial effect on aging in fruit flies, there is a lack of evidence that apoD contributes to lifespan expansion in mammals. It is noteworthy that apoD is abundantly expressed in the brain. Elderly subjects and patients with Alzheimer are associated with markedly elevated apoD production in the brain [72-74], but the underlying pathophysiology is unknown. It is important to understand whether and how apoD affects aging and contributes to lifespan expansion in mammals.

## Impact of apoD on atherosclerosis

**Figure 5.**
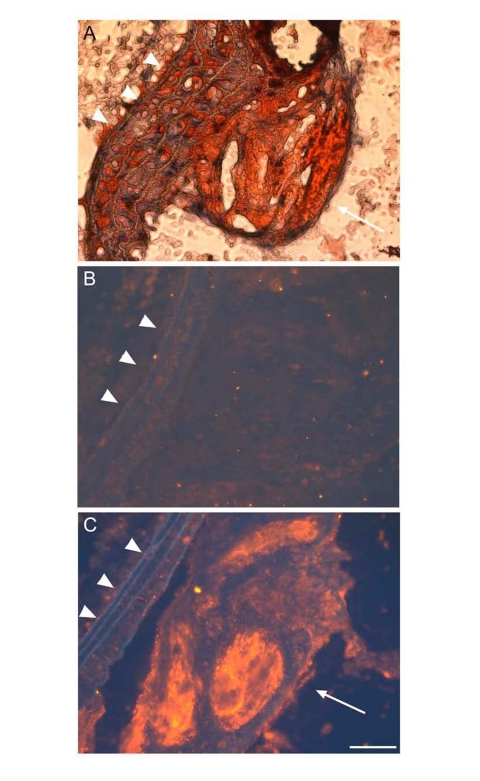
ApoD is localized to atherosclerotic plaques ofapoE?deficient mice. Proximal aorta sections of male apoE knockout mice were subjected to oil red O staining **(A)**, and to immunohistochemistry using control rabbit IgG against bacterial β-galactosidase **(B)** and rabbit anti-apoD antibody **(C)**. The secondary antibody used in immunostaining is the donkey anti-rabbit IgG conjugated with Cy3. ApoD was stained red in theatherosclerotic plaque **(C)**, as indicated by arrow. Elastic fibers ofvessels were auto-fluorescent blue, as indicated by arrowhead. Bar = 50 μm.

Does apoD contribute to atherosclerosis? To address this issue, Sarjeant et al [75] subject thin-sections of coronary arteries of archived human specimens to anti-apoD immunohistochemistry. Their studies visualize an increased apoD deposition in atheromatous plaques. Consistent with this finding, we show that apoD is localized in atherosclerotic lesions of apoE knockout mice (Figure 5). Thus, elevated apoD deposition along with excessive cholesterol accumulation is detectable in atherosclerotic lesions of both human and rodent origins. This is correlated with the ability of apoD to bind and transport cholesterol, raising the possibility that apoD may play a significant role in the pathogenesis of atherosclerosis. It follows that an increased deposition of apoD in atherosclerotic lesions can derive from a compensatory response of apoD to facilitate cholesterol removal from peripheral cells or result from the consequence of defects in apoD-mediated cholesterol trafficking. Further studies are warranted to distinguish whether apoD contributes to or protects against the development of atherosclerosis.

## Conclusions and perspectives

ApoD is a 29-kDa glycoprotein of 169 amino acids. ApoD is evolutionally conserved among species and is expressed in a variety of mammalian tissues. Although classifed as apolipoprotein, apoD belongs to the lipocalin family due to its structural adoptation of a β-barrel structure that is characteristic of lipocalins [40,41]. ApoD is shown to be a multi-ligand binding protein that is capable of transporting small hydrophobic molecules such as arachidonic acid, steroid hormones, and cholesterol for metabolism or signaling [34]. Altered apoD expression has been associated with a number of pathological conditions, including breast carcinoma, prostate cancer, Parkinson's disease, Alzheimer, schizophrenia, bipolar disorder, etc [13-17,34,72,74,76-80]. Elevated apoD deposition is also detected in amyloid plaques in the brains of patients with Alzheimer with undefined pathophysiology [15,79]. These data underscore the importance of apoD in the pathophysiology of cancer and neurological disorders. However, a comprehensive survey of apoD function is beyond the scope of this article. Instead, we center our review on apoD in lipid metabolism in relation to the pathogenesis of dyslipidemia and atherosclerosis, the two intertwined pathological traits that consequently predispose an at-risk individual to CAD.

ApoD is categorized as apolipoprotein due to its initial isolation from human HDL. Indeed, circulating apoD is bound mainly to HDL, correlating with the ability of apoD to associate via covalent cross-link with apoA-II. Plasma apoD is also present at a relatively low content in VLDL and LDL, suggesting that apoD plays significant roles in both triglyceride and cholesterol metabolism. Consistent with this notion, apoD polymorphism is associated with lipid disorders, as characterized by elevated plasma triglyceride levels and/or reduced HDL levels. ApoD is enriched in HDL isolated from patients with established CAD. Likewise, increased apoD deposition is detected in the atherosclerotic plaques of both human and rodent origins. However, a cause and effect relationship between aberrant apoD production and abnormal lipoprotein metabolism remains unknown. For example, how do apoD mutations result in elevated plasma triglyceride levels? Does apoD affect hepatic VLDL production and plasma VLDL clearance? Does apoD protect against or contribute to the pathogenesis of atherosclerosis? While apoD is present in HDL in dimerization with apoA-II, it is not clear how this inter-molecular cross-link affects HDL remodeling and impacts cholesterol metabolism. Obviously, further studies are needed to characterize the role of apoD in triglyceride and cholesterol metabolism and decipher the underlying mechanism that links apoD dysregulation to abnormalities in lipoprotein metabolism, accounting for heightened risk of developing CAD in subjects with obesity and/or diabetes.

Equally important, apoD is implicated to play a significant role in aging, as elevated apoD production results in lifespan extension in Drosophila. Elevated apoD production is seen in aging brains and altered brain apoD expression is associated with neurological disorders. It is of paramount importance to define apoD function in the brain and understand the molecular basis by which apoD affects aging and contributes neurological diseases.

## Materials and methods

Analysis of apoD N-glycosylation. Aliquots of plasma (20 μg protein) from C57BL/6J mice (male, 10 weeks old) were incubated without (-) and with (+) 1,000 U of N-glycosidase F (New England Biolabs) in a total volume of 30 μl at 37°C for 1 hour. The reaction mixture was resolved on 4-20% SDS-polyacrylamide gels, followed by immunoblot analysis using polyclonal rabbit anti-apoD (developed in our own laboratory).

RNA isolation and RT-PCR assay. Total RNA isolation from tissue (20 mg) was performed using the RNeasy Mini Kit (QIAGEN, Valencia, CA). Aliquots of purified RNA (100 ng) from were subjected to RT-PCR analysis using apoD sequence-specific primersflanking the apoD mRNA for forward reaction (5'-TAAGGCCTCTCCTGCAGCCA-3') and reverse reaction (5'-CTTTACAGGAAGTCCGGGCAG-3'). The resulting PCR products were resolved on 1% agarose gel containing ethidium bromide and visualized by UV light.

Immunohistochemistry. Mice were sacrificed and the proximal aorta of individual mice was dissected free of adipose and connective tissue, and immediately fixed in 4% paraformaldehyde. The aorta was mounted in Cryomatrix (Shandon, Pittsburgh, PA) and frozen in isopentane that has been pre-cooled in liquid nitrogen. Transverse cryo-sections (10 μm) were cut and stained by oil red O to visualize the atherosclerotic lesions. Consecutive sections were immunostained using either rabbit control IgG derived against bacterial β-galactosidase or polyclonal rabbit anti-apoD, followed by incubation with the donkey anti-rabbit IgG conjugated with Cy3. All animal studies were approved by the IACUC of Children's Hospital of Pittsburgh (protocol #30-07).

## Acknowledgement

This study was supported in part by American Diabetes Association and National Health Institute grant DK066301. We thank Dr. Steve Ringquist and members of the Dong Lab for critical proofreading of this manuscript.
